# *N*-Acetylcysteine (NAC) in Schizophrenia Resistant to Clozapine: A Double-Blind, Randomized, Placebo-Controlled Trial Targeting Negative Symptoms

**DOI:** 10.1093/schbul/sbac065

**Published:** 2022-07-20

**Authors:** Erica Neill, Susan L Rossell, Caitlin Yolland, Denny Meyer, Cherrie Galletly, Anthony Harris, Dan Siskind, Michael Berk, Kiymet Bozaoglu, Frances Dark, Olivia M Dean, Paul S Francis, Dennis Liu, Andrea Phillipou, Jerome Sarris, David J Castle

**Affiliations:** Centre for Mental Health, Faculty of Health, Arts & Design, Swinburne University of Technology, Melbourne, Australia; Department of Mental Health, St Vincent’s Hospital, Melbourne, Australia; Department of Psychiatry, University of Melbourne, Melbourne, Australia; Centre for Mental Health, Faculty of Health, Arts & Design, Swinburne University of Technology, Melbourne, Australia; Department of Mental Health, St Vincent’s Hospital, Melbourne, Australia; Centre for Mental Health, Faculty of Health, Arts & Design, Swinburne University of Technology, Melbourne, Australia; Department of Mental Health, St Vincent’s Hospital, Melbourne, Australia; Centre for Mental Health, Faculty of Health, Arts & Design, Swinburne University of Technology, Melbourne, Australia; Department of Psychiatry, Adelaide Medical School, University of Adelaide, Adelaide, Australia; Ramsay Health Care (SA) Mental Health, Adelaide, Australia; Northern Adelaide Local Health Network, Adelaide, Australia; Specialty of Psychiatry, Sydney Medical School, University of Sydney, Sydney, Australia; Westmead Institute for Medical Research, Westmead, NSW 2145, Australia; Metro South Addiction and Mental Health Service, Brisbane, Australia; UQ School of Clinical Medicine, Brisbane, Australia; Deakin University, IMPACT—the Institute for Mental and Physical Health and Clinical Translation, School of Medicine, Barwon Health, Geelong, Australia; Orygen, The National Centre of Excellence in Youth Mental Health, Centre for Youth Mental Health, Melbourne, Australia; Florey Institute for Neuroscience and Mental Health and the Department of Psychiatry, The University of Melbourne, Melbourne, Australia; Department of Paediatrics, The University of Melbourne, Melbourne, Australia; Bruce Lefroy Centre for Genetic Health Research, Murdoch Children’s Research Institute, Melbourne, Australia; Metro South Addiction and Mental Health Service, Brisbane, Australia; UQ School of Clinical Medicine, Brisbane, Australia; Deakin University, IMPACT—the Institute for Mental and Physical Health and Clinical Translation, School of Medicine, Barwon Health, Geelong, Australia; Orygen, The National Centre of Excellence in Youth Mental Health, Centre for Youth Mental Health, Melbourne, Australia; School of Life and Environmental Sciences, Faculty of Science, Engineering and Built Environment, Deakin University, Waurn Ponds, VIC, Australia; Department of Psychiatry, Adelaide Medical School, University of Adelaide, Adelaide, Australia; Northern Adelaide Local Health Network, Adelaide, Australia; Centre for Mental Health, Faculty of Health, Arts & Design, Swinburne University of Technology, Melbourne, Australia; Orygen, The National Centre of Excellence in Youth Mental Health, Centre for Youth Mental Health, Melbourne, Australia; NICM Health Research Institute, Western Sydney University, Westmead, NSW, Australia; Professional Unit, The Melbourne Clinic, Department of Psychiatry, University of Melbourne, Melbourne, VIC, Australia; Centre for Mental Health, Faculty of Health, Arts & Design, Swinburne University of Technology, Melbourne, Australia; Department of Mental Health, St Vincent’s Hospital, Melbourne, Australia; Department of Psychiatry, University of Melbourne, Melbourne, Australia

**Keywords:** mental illness, quality of life, cognition, depression, mental disorders, psychiatry, neuroscience

## Abstract

**Background and Hypothesis:**

Clozapine is the most effective antipsychotic for treatment-resistant schizophrenia, yet a significant proportion of individuals on clozapine continue to experience disabling symptoms, despite being treated with an adequate dose. There is a need for adjunct treatments to augment clozapine, notably for negative and cognitive symptoms. One such potential agent is the glutathione precursor *N*-acetylcysteine (NAC).

**Study Design:**

A randomized double-blind, multi-center, placebo-controlled trial for clozapine patients with enduring psychotic symptoms (*n* = 84) was undertaken to investigate the efficacy of adjunctive NAC (2 g daily) for negative symptoms, cognition and quality of life (QoL). Efficacy was assessed at 8, 24, and 52 weeks.

**Study Results:**

NAC did not significantly improve negative symptoms (*P* = .62), overall cognition (*P* = .71) or quality of life (Manchester quality of life: *P* = .11; Assessment of quality of life: *P* = .57) at any time point over a 1-year period of treatment. There were no differences in reported side effects between the groups (*P* = .26).

**Conclusions:**

NAC did not significantly improve schizophrenia symptoms, cognition, or quality of life in treatment-resistant patients taking clozapine. This trial was registered with “Australian and New Zealand Clinical Trials” on the 30 May, 2016 (Registration Number: ACTRN12615001273572).

## Introduction

Schizophrenia is characterized by positive (eg, hallucinations, delusions), disorganized (eg, disorganized thoughts and behavior), negative (eg, anhedonia and avolition), and cognitive symptoms (eg, poor attention and memory). While the positive symptoms are usually adequately managed with antipsychotics, negative symptoms respond less, and cognitive symptoms often remain resistant to treatment.^1–3^ Furthermore, the cognitive and negative symptoms are the greatest contributors to poor quality of life (QoL) and reduced daily functioning in people with schizophrenia. Treatment resistant (TR) schizophrenia describes a failure to respond to two adequate trials of first-line antipsychotics^4^ and affects 25%–33% of people with schizophrenia.^5^ Clozapine is the most effective antipsychotic for reducing positive psychotic symptoms^6^ and hospitalizations^7^; however, around 40% of individuals do not achieve adequate response, even with a therapeutic dose of clozapine.^8^ There is a paucity of effective augmenting agents to enhance clozapine response, particularly for residual negative and cognitive symptoms.^9,10^

Numerous augmentation strategies have been used to boost the efficacy of clozapine for those with ongoing symptoms.^9–11^ These include pairing clozapine with other antipsychotics, and adding antidepressants, mood stabilizers, or glutamatergic agents.^9,12^ At this stage, the benefits of these augmentation strategies are under-researched, but appear to be minimal in impact, or in some cases detrimental, due to drug interaction effects and added side-effect burden.^13^ Any agent taken adjunctively with clozapine that might enhance efficacy, without adding to the side effects, could improve the lives of people with clozapine refractory schizophrenia.^14,15^ One such potential agent is *N*-acetylcysteine (NAC), a precursor to glutathione (GSH), which modulates both glutamate and dopamine, and reduces oxidative stress and inflammation.^16^ In schizophrenia, decreased levels of GSH have been identified through magnetic resonance imaging, genetic and cerebrospinal fluid studies.^17–19^

The first study of NAC for schizophrenia^17^ found a statistically significant effect for NAC over 24 weeks, particularly in negative symptoms; almost half of those successfully treated with NAC were taking clozapine. This raised the question of whether NAC might be effective in clozapine patients with residual symptoms. A subgroup investigation^17^ found that there was a statistically significant improvement in negative and total PANSS scores after 8 weeks of treatment in those taking clozapine, supporting a potential role for NAC in this group.^20^ To the authors’ knowledge, this is the only study to have investigated the potential for NAC to improve outcomes for those on clozapine specifically.

A recent meta-analysis synthesized double-blind placebo-controlled trials of NAC in schizophrenia and first episode psychosis.^21^ Seven studies met inclusion criteria and provided a measure of psychosis using the PANSS.^22^ Meta-analytic findings suggested that NAC improved PANSS symptoms after 24 weeks of treatment with a large effect for both negative (standardized mean difference (SMD) −0.72, *P* = .003) and total PANSS scores (SMD −0.92, *P* < .001). The improvements in negative symptoms were of note given their resistance to current antipsychotic medication options, including clozapine.

In addition to improvements in negative symptoms, there is reason to believe that NAC may improve cognition in schizophrenia. Firstly, decreased GSH and oxidative stress are implicated in the cognitive decline associated with both normal aging and neurodegenerative disorders.^23^ Secondly, reduced GSH has been associated with depletion in brain-derived neurotrophic factor (BDNF) and *N*-methyl-d-aspartate (NMDA) hypo-function, both of which are associated with cognitive impairment. Finally, NAC improves glutamate function, a pathway that is important for normal cognitive processing.^23^ Evidence that NAC can improve cognition in schizophrenia comes from case studies^24^ and a number of clinical trials.^25–27^ Of the clinical trials that examined cognition, 2 found that processing speed was improved by NAC^25,27^ and 2 that working memory was improved^26,27^; while one found no effect of NAC on cognition.^28^ Previous trials of glutamatergic agents in schizophrenia have often provided disappointing results.^29,30^ In the case of NAC, however, in addition to its impact on the glutamatergic system, it has other relevant mechanisms of action, including redox anti-inflammatory pro neurogenesis and enhanced mitochondrial energy generation which set it apart from previously tested agents.

If NAC can improve both negative symptoms and cognition in people with schizophrenia, then it is likely that it will also improve QoL. To date, we are aware of no published data examining whether this relationship exists in schizophrenia, but there are data to support a positive effect of NAC on QoL in people with bipolar disorder.^31^ This same bipolar trial was focused on the impact of NAC on mood and found an improvement in depression after treatment with NAC. A further follow-up trial by this same group^32^ confirmed the utility of NAC in improving mood in bipolar II. As such, in the study reported here, a follow-up exploratory analysis (not included in the initial protocol^33^) is included to examine whether this same improvement in mood might be found in TR schizophrenia.

In the context of small studies and secondary outcomes, there is sufficient evidence to suggest that a full-scale, long-term trial of adjunctive NAC for people with TR schizophrenia currently taking clozapine, is warranted. We report a 52-week randomized placebo-controlled trial of NAC (2 g daily) vs placebo in people with schizophrenia who were stabilized on clozapine but experienced residual symptoms. Our primary hypothesis was that in a group of TR schizophrenia patients, NAC would improve negative symptoms compared with the placebo group. Our two secondary hypotheses were that NAC would improve cognition and QoL. The final exploratory hypothesis is that NAC may improve symptoms of depression.

## Methods

### Study Design

**Figure 1.**
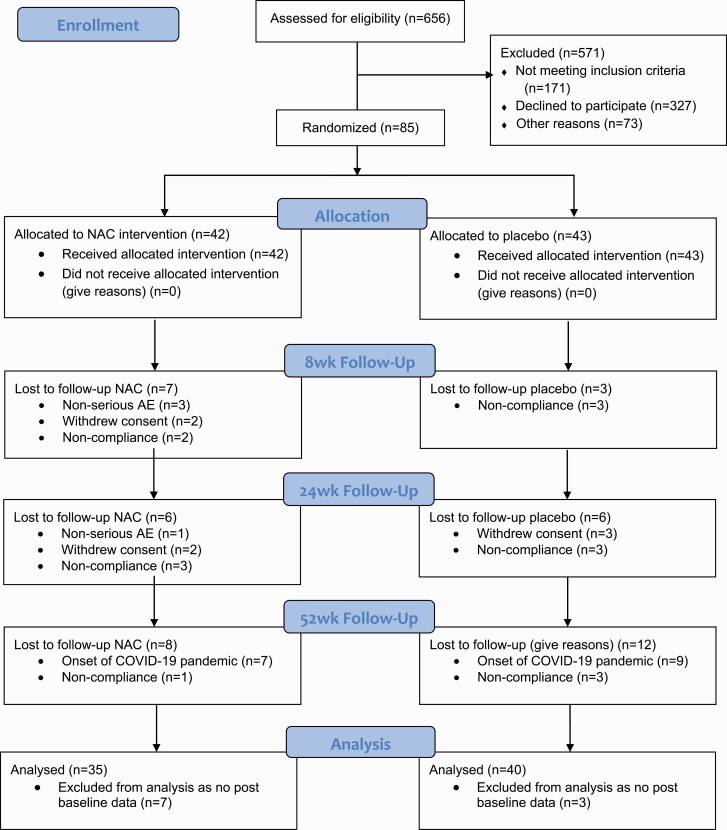
CONSORT diagram.

In this randomized, double-blind, placebo-controlled trial, participants were allocated to 2 g/day NAC or placebo with assessments at 0, 8, 24, and 52 weeks (figure 1). Participants remained on all of their usual medications throughout their participation in the trial, including antipsychotic drugs. Data for this trial were collected between March 2017 and March 2020. Participants were referred by clinicians or recruited from preexisting research databases. Study sites included public hospitals and outpatient facilities in the Australian cities of Melbourne, Brisbane, Adelaide, and Sydney. The protocol has been published elsewhere.^33^ This trial was registered with the Australian and New Zealand Clinical Trials Registry on the 30 May 2016 (Registration Number: ACTRN12615001273572), was funded by the National Health and Medical Research Council project grant (NHMRC APP1098442) and received ethical approval. Study data were collected and managed using REDCap electronic data capture tool hosted at The University of Melbourne. For information relating to small deviations from the protocol^33^ see Supplementary material.

### Inclusion/Exclusion Criteria

Participants were required to meet Diagnostic and Statistical Manual of Mental Disorders, Fifth Edition (DSM-5)^34^ criteria for either schizophrenia or schizoaffective disorder, were between 18 and 65 years of age, and able to give informed consent. All participants provided written informed consent. Participants were required to have had at least three periods of treatment in the preceding 5 years with antipsychotic drugs from different chemical classes without significant symptomatic relief. In addition, they were on a stable dose of clozapine for at least 6 months, and, despite adequate dosing (serum level of >350 µg/L) they continued to experience residual symptoms, defined as either a score of >4 on two or more PANSS negative items or a total PANSS score ≥60. Clozapine dose and blood levels were confirmed by referring physician or online medical records. Finally, females of childbearing age who were sexually active were required to be using adequate contraception. Participants were excluded if they were already taking NAC or were known to be allergic to it. Further exclusions included those taking nitro-glycerin, aralen, or selenium. Those who had diabetes mellitus, had a known primary or secondary autoimmune disorder, a history of gastrointestinal ulcers or kidney stones, were pregnant or were enrolled in another intervention study were also excluded.

### Intervention

Randomization was performed by a researcher independent of the trial using a block randomization (2 × 4) with a 1:1 sequential allocation. On request, local investigators received a number to allocate to each participant. The key linking treatment bottles with treatment arms was maintained by the independent researcher until data collection was complete. An emergency code break envelope was held by the principal investigator.

Participants in the active condition received two 500 mg NAC capsules to take twice daily, resulting in a total daily dose of 2 g. Dividing the dose into morning and evening reduced the likelihood of gastrointestinal upset (a potential side effect) and improved consistency of NAC’s impact (given its short half-life). Placebo capsules were manufactured identically and, in addition, the desiccant sachet included in the placebo bottles were scented to match that of the NAC capsules, which have a sulfurous smell. The NAC and placebo were provided by BioCeuticals Australia, and they were responsible for encapsulating the products and sealing the bottles. Participants were provided with sufficient capsules to last until the next scheduled visit, plus approximately 3 days extra. Capsules were counted at each visit so that adherence could be determined. Participants were excluded if they missed ≥50% of their NAC/placebo doses at any assessment point.

### Assessments

Participant interviews, consultation with participant psychiatrists, and medical notes were used for initial assessment of inclusion/exclusion criteria. This was followed up by trained researchers confirming diagnoses using the SCID-5.^34^ Participants who were eligible after screening were enrolled by site investigators.

The primary outcome was the negative symptom subscale of the PANSS.^22^ The PANSS is the most commonly used symptom rating scale for schizophrenia clinical trials.^35^ Secondary outcomes explored positive and general symptoms, cognition, QoL, and mood. Cognitive performance was assessed using the MATRICS consensus cognitive battery (MCCB).^36^ The MCCB is the gold standard measure for cognition in clinical trials for schizophrenia.^37^ It provides seven domain scores (eg, memory, attention) and an overall cognition score, providing a more in-depth investigation of cognition than has been employed in earlier NAC schizophrenia trials. QoL was measured using the Manchester short assessment of quality of life (MANSA)^38^ and the assessment of quality-of-life (AQoL)^39^ scales providing both objective and subjective measures of QoL. The very brief psychosis treatment scale side-effect module^40^ was included to determine whether the groups differed in extra pyramidal side effects. Finally, mood symptoms were assessed using the calgary depression scale for schizophrenia (CDS)^41^ and the depression subscale of the PANSS.^42^ The depression subscale is made up of three PANSS items assessing guilt, anxiety, and sadness/depression. In addition to examining the impact of NAC on depression, depression severity was assessed so it could be accounted for in the negative symptom mixed model repeated measures analyses (using the CDS specifically).

Assessments were conducted at 0, 8, 24, and 52 weeks. The 52-week assessment extends this trial 6 months longer than the longest previous trial (at the time the protocol was devised in 2016).^21^ This extension to a year was based on the observation from a previous trial that NAC improvements were taking place but slowly, potentially requiring a longer treatment before significant changes could be seen.^17^ Between visits, participants were contacted weekly to assess study medication adherence and safety using the systematic assessment for treatment emergent events (SAFTEE).^43^ The SAFTEE was designed for use in clinical trials and assesses a wide range of symptoms including affective, behavioral, and somatic symptoms. It was designed to prevent underreporting of symptoms in clinical trials.

### Statistical Analyses

The power analysis assumed one primary outcome measure (PANSS Negative scores), four assessment points (0, 8, 24, and 52 weeks), a study-wide type I error rate (α) of .01, a type II error rate (β) of .10 (power of .90), a correlation of post-treatment scores with baseline measurements (ρ) of 0.70, and a two-tailed statistical test. To detect a medium effect size of *d* = 0.5 as based upon previous studies,^17^ 45 participants in each of the study arms would ensure adequate power. This corresponds to an η^2^ value of 0.06.^44^

Data were analyzed using SPSS v27. Scales were computed for the outcome measures using the expectation-maximization algorithm to impute missing items. Potential group differences in dropout rates and medication adherence were examined using Chi-square analysis. The groups were compared in terms of demographics and baseline psychosis symptoms using nonparametric tests (Fisher exact tests for categorical and Mann–Whitney tests for continuous variables) due to non-normal distributions of some scales. Binary logistic regression analyses were used to determine whether attrition at 8, 24, or 52 weeks could be predicted using any study, demographic, or baseline psychosis symptom variables. The only variable associated with attrition was “site”, and this was controlled for in all ensuing analyses.

Transformations were necessary for the CDS (logarithmic) and SAFTEE (square transformations) measures. Furthermore, correlations between the outcome measures and variances at assessment time points were similar, making the assumption of compound symmetry appropriate for the residual covariance matrix. A mixed-model repeated-measures (MMRM) analysis was conducted to address the hypotheses for the primary, secondary, and exploratory outcome measures using the four assessment time points. Intention-to-treat analyses with fixed effects for treatment, site, time, and the treatment × time interaction were conducted. No group differences were identified in demographic or baseline psychosis symptom analyses (table 1), thus no covariates were applied to the MMRM. In the case of the primary hypothesis, PANSS positive and PANSS depression were controlled for in the analyses, as they can impact negative symptoms both directly and indirectly.^45^

**Table 1.** Demographics by group.

	NAC	Placebo	Sig.
Male (*n*, %)	28 (66.7%)	33 (76.7%)	0.39
Years of education^*^	12.92 (2.61)	12.77 (3.09)	0.82
Age at testing^*^	39.83 (9.19)	39.65 (9.41)	0.98
Age at onset of symptoms^*^	24.12 (7.15)	23.87 (6.40)	0.88
Duration of illness since symptom onset^*^	19.06 (11.07)	17.70 (9.10)	0.89
Clozapine level ng/ml^*^	543.15 (270.14)	541.83 (249.20)	0.98
Total number of medications^*^	4.61 (2.80)	4.18 (2.58)	0.38
PANSS negative^*^	20.14 (5.52)	18.32 (5.39)	0.60
PANSS positive^*^	16.40 (6.55)	15.89 (5.44)	0.60
PANSS general^*^	33.89 (8.34)	33.55 (7.00)	0.47
SAFTEE score^*^	31.56 (18.59)	36.82 (20.96)	0.26
Extrapyramidal symptoms^*^	2.00 (2.62)	1.38 (1.97)	0.25
Schizophrenia/Schizoaffective diagnosis	27/2	30/3	0.93

*Note*:

Data presented are mean (standard deviation); PANSS: positive and negative symptom syndrome; SAFTEE: systematic inquiry about emergent clinical events.

## Results

### Descriptive Statistics

Of 656 people who were screened, 85 were included in the study, with 42 assigned to NAC and 43 to placebo (see figure 1). The numbers of participants recruited at each site were 10 (11.8%) for Adelaide, 15 (17.6%) for Brisbane, 26 (30.6%) for Melbourne and 34 (40.0%) for Sydney. No statistically significant differences were observed between the two groups for demographic or clinical variables at baseline. There were no differences in the number of antipsychotics (*χ*^2^ = 2.37, *P* = .31), antidepressants (*χ*^2^ =2.94, *P* = .09) or mood stabilizing medications (*χ*^2^ =0.38, *P* = .54) taken by the participants in either group. Neither dropout rates (*P* = 0.71) nor medication adherence rates (*P* = 0.37) differed between the NAC and placebo group.

The completion rate for the 8-week (*n *= 72, 84.9%), 24-week (*n *= 60, 70.9%), and 52-week (*n *= 41, 47.7%) assessments was similar for the two groups (*P *= .362, *P *= .379, and *P *= 1.000, respectively). However, attrition rates at 24 weeks differed significantly between the sites (*χ*^2^ = 14.73, df = 3, *P *= .002) with the highest attrition rate for Melbourne (46.2%) followed by Adelaide (40.0%), then Sydney (23.5%), with no attrition for Brisbane. Thus site was controlled for in the ensuing analyses. No other baseline variables were significantly related to attrition at week 8, week 24, or week 52. The number of prescribed medications also differed significantly between the sites (*χ*^2^ = 23.59, df* = *3, *P *< .001), with the highest mean number of medications for Brisbane (MN = 7.13, SE = 0.78), compared to lower mean numbers for Adelaide (MN = 4.38, SE = 0.84), Melbourne (MN = 3.75, SE = 0.45), and Sydney (MN = 3.69, SE = 0.38).

### Mixed-Model Repeated-Measures (MMRM) for Outcome Measures

**Figure 2.**
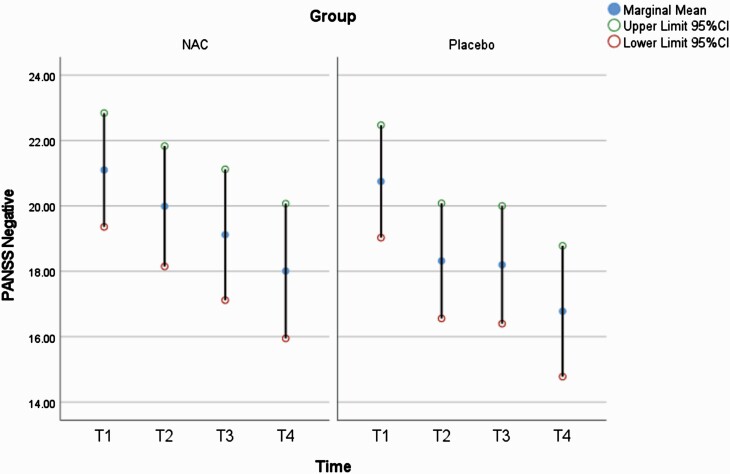
PANSS negative scores at each assessment.

The MMRM analyses are presented throughout this section and in table 2 (primary and secondary outcomes are highlighted in gray). For our primary outcome of PANSS negative, the time effect was significant (*F*(3,180) = 12.59, *P *< .001), but there was no significant group × time interaction (*F*(3,180) = 0.60, *P *= .616) (see table 2 for full MMRM results; see figure 2).

**Table 2.** MMRM analyses for outcome measures for T1–T4.

Outcomes	Marginal means (Std errors)^*^								Group effect		Time effect		Time × Group effect		
	NAC				Placebo										
	T1 *n* = 42	T2 *n* = 35	T3 *n* = 29	T4 *n *= 21	T1 *n* = 43	T2 *n* = 40	T3 *n* = 34	T4 *n* = 22	*F*(1,*df*)	*p*	*F*(3,*df*)	*p*	*F*(3,*df*)	*p*	η^2^
Symptoms (score range)															
PANSS negative(7-49)	21.10 (0.87)	19.99 (0.92)	19.12 (1.00)	18.01(1.03)	20.75 (0.86)	18.32 (0.88)	18.20 (0.90)	16.78 (1.00)	0.98	.325	12.59	**<.001**	0.60	.616	.003
PANSS positive(7-49)	17.34 (0.97)	16.27 (1.00)	16.28 (1.04)	15.07 (1.10)	18.37 (0.95)	15.81 (0.96)	15.50 (0.99)	14.85 (1.07)	0.01	.928	10.14	**<.001**	1.28	.282	.003
PANSS General (16-112)	34.81 (1.17)	33.42 (1.25)	34.28 (1.32)	31.27 (1.46)	36.50 (1.15)	33.76 (1.19)	34.07 (1.23)	33.55 (1.42)	0.58	.449	4.29	**.006**	0.76	.521	.005
PANSS depression (3-21)	7.20 (0.49)	6.23 (0.51)	6.83(0.53)	5.92 (0.57)	8.15 (0.48)	7.40 (0.49)	7.19 (0.50)	8.08 (0.55)	3.73	.057	4.21	**.007**	2.70	**.047**	**.012**
Calgary Depression(0-27)	1.63 (1.15)	1.01 (1.15)	1.69 (1.16)	1.09 (1.17)	2.18 (1.15)	2.66 (1.15)	2.79 (1.15)	3.08 (1.17)	7.23	**.009**	1.32	.269	3.38	**.020**	**.012**
Extrapyramidal Symptoms (0-26)	1.78 (0.34)	1.47 (0.37)	1.00 (0.40)	0.30(0.45)	1.21 (0.33)	1.07 (0.34)	1.51 (0.36)	0.89 (0.44)	0.01	.920	1.99	.118	1.45	.229	.019
MATRICS *T*-scores															
OvComp	25.25 (2.13)	30.57 (2.18)	31.00 (2.27)	31.03 (2.33)	25.29 (2.01)	29.16 (2.03)	30.70 (2.07)	29.09 (2.24)	0.11	.737	16.13	**<.001**	0.46	.710	.006
PS	34.76 (2.10)	36.72 (2.17)	37.62 (2.24)	37.45 (2.35)	34.78 (2.08)	37.42 (2.10)	37.94 (2.15)	38.47 (2.32)	0.04	.847	4.09	**.008**	0.08	.971	.004
ATT	30.05 (2.14	35.69 (2.22)	33.14 (2.29)	32.12 (2.44)	31.06 (2.05)	35.37 (2.08)	36.24 (2.14)	34.20 (2.40)	0.32	.573	7.56	**<.001**	0.73	.537	.005
WM	35.12 (2.02)	34.76 (2.07)	36.89 (2.14)	36.07 (2.24)	35.33 (2.00)	37.69 (2.01)	38.68 (2.06)	37.16 (2.20)	0.35	.558	2.51	.061	0.84	.471	.003
VerL	28.72 (1.25)	32.63 (1.32)	34.00 (1.41)	34.65(1.54)	29.57 (1.24)	32.84 (1.27)	33.90 (1.33)	32.85 (1.52)	0.02	.887	12.65	**<.001**	0.58	.631	.005
VisL	29.17 (2.43)	36.21 (2.53)	34.02 (2.63)	38.61 (2.76)	32.62 (2.40)	35.68 (2.44)	38.26 (2.50)	36.41 (2.72)	0.17	.684	10.21	**<.001**	2.50	.061	.006
RaPs	40.35(1.60)	43.25 (1.69)	41.84 (1.77)	41.46 (1.90)	38.24 (1.59)	39.34 (1.62)	42.06 (1.67)	39.75 (1.87)	0.93	.337	2.76	**.044**	1.39	.247	.003
SC	34.11 (2.27)	36.88 (2.35)	37.30 (2.52)	35.72 (2.58)	36.45 (2.19)	37.79 (2.22)	35.87 (2.29)	37.87 (2.48)	0.12	.725	1.13	.340	0.88	.452	.008
Safety/Side effects measure															
SAFTEE (SQRT)	5.56 (.323)	4.73 (.329)	4.63 (.336)	4.40 (.370)	5.86 (.311)	5.37 (.311)	5.27 (.319)	5.33 (.345)	2.47	.120	11.11	**<.001**	1.04	.379	.008
Quality of life measures															
MANSA QoL	55.44 (1.94)	59.58 (2.10)	54.68 (2.27)	59.64 (2.49)	54.54 (1.89)	57.24 (1.99)	57.92 (2.06)	55.37 (2.36)	0.98	.325	2.43	.067	2.04	.111	.019
AQoL	20.06 (0.70)	18.00 (0.72)	18.86 (0.75)	18.70 (0.80)	20.21 (0.68)	19.14 (0.70)	19.58 (0.71)	18.90 (0.78)	0.43	.514	5.79	**.001**	0.67	.569	.003

*Note*: Grey rows relate to primary and secondary outcomes. Data in bold refer to significant differences. T1: baseline, T2: 8 weeks, T3: 24 weeks, T4: 52 weeks, *df*: degrees of freedom. OvComp (overall composite), PS (processing speed), ATT (attention/vigilance), WM (working memory), VerL (verbal learning), VisL (visual learning), RaPs (reasoning and problem solving), SC (social cognition), SAFTEE (SQRT) systematic inquiry about emergent clinical events–square root, MANSA QoL (Manchester short assessment of quality of life), AQoL (assessment of quality of life).

Marginal means and standard error reported as controlling for study site.

For the secondary outcome of cognition, we examined the MCCB global score, in addition to exploring any effects on the seven domains. As illustrated in table 2, there were time effects for the global score and all domains except working memory and social cognition. There were no significant group × time interactions for the global score or seven domain scores.

Our secondary outcomes relating to QOL, MANSA, and AQoL, did not show group × time interactions, with only AQoL showing a time effect (see table 2). Further exploratory MMRM showed improvement for the CDS, with a significant interaction effect for group × time (*F*(3,177) = 3.38, *P* = .020, η^2^=.012) in favor of the NAC group and significantly higher scores for the NAC group than the placebo group overall (*F*(3,179) = 2.70, *P* = .047, η^2^ = .059). In addition there was a significant interaction effect for group × time on the depression component of the PANSS (*F*(3,179) = 2.70, *P* = .047, η^2^=.012) (see Supplementary figure 1). However, when the 6 participants with changes in their clozapine dosage were removed this result was not significant (*F*(3,173) = 2.04, *P* = .111). It should be noted that when alternative measures of the PANSS depression scale including that proposed by Lindenmayer et al^46^ and Lancon et al,^47^ these results did again not reach significance; *P* = 0.06 in both cases (see Supplementary table 1), with *P* = 0.09 and *P* = 0.11, respectively, when the 6 participants with changes in their clozapine dosage were removed. For no other measure were there changes in the conclusions when the 6 people who recorded changes in clozapine dosage were removed. This is not surprising because the 3 participants in each of the groups were well matched according to their changes in dosage.

There were no significant time, group, or group × time interaction effects for the measure of participant safety (SAFTEE) indicating no increase in side effects over the 52 weeks of this trial (see table 2). Further, there was no difference in medication adherence between the 2 groups (*P* = .54).

## Discussion

The current study describes the first RCT of NAC specifically for clozapine-treated TR schizophrenia. The results do not support any efficacy of adjunctive NAC (2 g/day) over 52 weeks in improvement of negative symptoms, cognition, or QoL in this group (after controlling for study site—the only factor associated with attrition). This finding contrasts earlier pilot data (placebo, *N* = 27, NAC, *N *= 28) which found an improvement in PANSS negative and total scores after 8 weeks of treatment with NAC.^20^ Compared to the participants in the pilot study, those in the current trial received the same dose of NAC, were similar in both age and length of illness at baseline, and in scores on PANSS positive, negative, and general subscales (see Supplementary table 2). It should be noted that while the pilot data did identify a significant improvement on the PANSS negative symptom subscale at 8 weeks, this improvement was not maintained at 24 weeks. In addition, the inclusion criteria for the current study required slightly higher PANSS scores for inclusion (score of >4 on two or more PANSS negative items or a total PANSS score ≥60) compared with the pilot study (score of 55 on PANSS or at least two positive and/or negative items being 3). Furthermore, the significant finding was of small effect (*d* = 0.3). As such, with a larger sample, this finding was not replicated at 8, 24, or 52 weeks.

With regard to cognitive outcomes, we chose to analyze overall cognition as well as the subscales of the MCCB. There was no effect of NAC on overall cognition or any cognitive domain, at any of the 3 assessment time points. Previous literature indicated that NAC may improve specific as opposed to global cognition, with some studies reporting improvements in working memory,^26^ processing speed,^25^ or executive function^27^; see Yolland et al review and Yolland et al (2020) meta-analysis for a more in-depth examination.^21,23^

Finally, it was hypothesized that, because of improvements in negative symptoms and cognition, there would also be improvements in subjective QoL. This hypothesis was also not supported by either of the QoL measures (MANSA or AQoL). Considering the non-significant results across negative symptoms and cognition, this finding is perhaps not surprising.

Previous NAC trials have demonstrated that adjunctive NAC may be useful in treating depression,^31,32^ and this possibility was explored using the current data. Our results indicate a statistically significant improvement in mood across two measures of depression (CDS and PANSS Depression). This exploratory finding should be interpreted with caution on the basis that, on both measures, this was a change of small effect (η^2^ =.01 for both measures). In addition, on a group level, participants did not have clinically meaningful depression scores at baseline, with a mean score on the CDS of 1.63 for the NAC group and 2.18 for the placebo group (scale range 0–27) (see Supplementary figures 1 and 2). Furthermore, the PANSS depression subscale can be calculated using several different methods (3 items,^42^ 4 items,^46^ or 5 items^47^) none of these methods generated significant findings (see Supplementary table 1). As such, as opposed to interpreting this as evidence that NAC has improved depression in TR schizophrenia patients, it is an indication that should be further investigated in a group of TR schizophrenia patients with comorbid depression.

### Strengths/Limitations

This is the first NAC trial to target those with clozapine resistant schizophrenia. It extends previous literature by increasing the length of treatment and the number of participants enrolled. In addition, an extensive battery of cognitive tests and measures of QoL were included. Limitations include the lower than expected recruitment rate, resulting in low final numbers (NAC = 21, placebo = 20). However, given the findings did not approach significance provides some assurance that the results are meaningful, despite the limited sample size. The data also confirm the good safety profile of NAC, with no differences in side effect burden between the groups at any time point. While the selected dose of 2 g a day has been associated with improvements in negative symptoms in previous schizophrenia trials,^21^ it may be that a larger dose of NAC (doses of up to 3600 mg are reported)^28^ is necessary in order to see sustained improvements, particularly for a TR group.

## Conclusion

The findings of this study do not support the efficacy of NAC for negative or cognitive symptoms, or for improvements in QoL for people with schizophrenia experiencing residual symptoms on clozapine. There is some exploratory evidence that depression may have improved as a result of NAC treatment, but this must be further investigated, preferably within a group with more significant depression symptoms.

## Supplementary Material

sbac065_suppl_Supplementary_Material

## Deviations from Protocol

Two assessments that were outlined in the protocol paper for this study (34) were removed including both the Clinical Assessment Interview for Negative Symptoms (CAINS) (44) and the Montgomery Asberg Depression Scale (MADRS) (45). This decision was made to reduce the length of the testing battery to ensure the comfort and compliance of participants.

After the publication of the protocol, but prior to the recruitment of participants, the members of the DSMB advised that the power analysis was too conservative. In the protocol, the power analysis outlined a power level of 0.9 and correlation at 0.7. In response, the original power was adjusted from 0.9 to 0.8 and the correlation from 0.7 to 0.5. As a result of these changes, a new total of 62 participants was required to get through to 52 weeks.
