# Supplementary material for: N-Acetylcysteine (NAC) in Schizophrenia Resistant to Clozapine: A Double-Blind, Randomized, Placebo-Controlled Trial Targeting Negative Symptoms
Source: Schizophr Bull. 2022 Jul 20;48(6):1263–72. doi: 10.1093/schbul/sbac065 (PMC9673271; doi:10.1093/schbul/sbac065)
Supplement: sbac065_suppl_Supplementary_Material [file sbac065_suppl_supplementary_material.docx]

**Deviations from Protocol**

Two assessments that were outlined in the protocol paper for this study (34) were removed including both the Clinical Assessment Interview for Negative Symptoms (CAINS) (44) and the Montgomery Asberg Depression Scale (MADRS) (45). This decision was made to reduce the length of the testing battery to ensure the comfort and compliance of participants.

After the publication of the protocol, but prior to the recruitment of participants, the members of the DSMB advised that the power analysis was too conservative. In the protocol, the power analysis outlined a power level of 0.9 and correlation at 0.7. In response, the original power was adjusted from 0.9 to 0.8 and the correlation from 0.7 to 0.5. As a result of these changes, a new total of 62 participants was required to get through to 52wks.

**Supplementary Table 1. Alternative Measures of PANSS Depression Compared**

|  | NAC | | | | Placebo | | | | Time Effect | | Time*Group Effect | | |
| --- | --- | --- | --- | --- | --- | --- | --- | --- | --- | --- | --- | --- | --- |
| Outcomes | T1 n=42 | T2 n=35 | T3 n=29 | T4 n=21 | T1 n=43 | T2 n=40 | T3 n=34 | T4 n=22 | F(3,df) | p | F(3,df) | p | η2 |
| 3 item PANSS Depression*  (Wallwork et al., 2012) | 7.20 (0.49) | 6.23 (0.51) | 6.83 (0.53) | 5.92 (0.57) | 8.15 (0.48) | 7.40 (0.49) | 7.19 (0.50) | 8.08 (0.55) | 4.21 | .007 | 2.70 | .047 | .012 |
| 4 item PANSS Depression (Lindenmayer et al., 1994) | 8.85 (0.57) | 8.31 (0.59) | 8.63 (0.62) | 7.65 (0.66) | 9.94 (0.56) | 9.44 (0.57) | 9.09 (0.58) | 10.26 (0.64) | 1.24 | .297 | 3.69 | .058 | .011 |
| 5 item PANSS Depression  (Lancon et al., 1998) | 11.07 (0.63) | 10.24 (0.66) | 10.71 (0.68) | 9.51 (0.73) | 11.68 (0.61) | 11.16 (0.63) | 11.11 (0.64) | 12.11 (0.71) | 1.37 | .253 | 2.46 | .064 | .011 |

**Used in main analysis*

*Wallwork et al., 2012: PANSS G1, G2, G6*

*Lindenmayer et al., 1994: PANSS G1, G2, G3, G6*

*Lancon et al., 1998: PANSS G1, G2, G3, G6, G15*

**Supplementary Table 2. Baseline Data Comparisons between Dean 2018 and Current Trial**

|  | **Dean (2018)** |  |  |  | **Current NAC trial** | | |  |
| --- | --- | --- | --- | --- | --- | --- | --- | --- |
|  | **Placebo Group** |  | **NAC Group** |  | **Placebo Group** |  | **NAC Group** |  |
| **PANSS *M (SD)*** |  |  |  |  |  |  |  |  |
| Positive | 16.67 (5.76) |  | 16.61 (5.38) |  | 15.89 (5.44) |  | 16.40 (6.55) |  |
| Negative | 15.00 (6.60) |  | 18.36 (6.14) |  | 18.32 (5.39) |  | 20.14 (5.52) |  |
| General | 31.52 (7.29) |  | 32.71 (9.71) |  | 33.55 (7.00) |  | 33.89 (8.34) |  |
| Total | 63.19 (15.05) |  | 67.68 (17.67) |  | 67.76 (14.25) |  | 70.43 (14.16) |  |

**Supplementary Figure 1. PANSS Depression over 52 weeks**

*Means and SD plotted*

**Supplementary Figure 2. Calgary Depression over 52 weeks**

*Means and SD plotted*
